# Taking a close look at electrosensing

**DOI:** 10.7554/eLife.16209

**Published:** 2016-04-29

**Authors:** Tatyana O Sharpee

**Affiliations:** Computational Neurobiology Laboratory, Salk Institute for Biological Studies, La Jolla, United Statessharpee@salk.edu

**Keywords:** *Apteronotus leptorhynchus*, weakly electric fish, electrosensory, neural coding, natural stimuli, Other

## Abstract

The brain of the brown ghost knifefish, which uses electric fields to “see”, processes electrical signals in a way that is similar to how our brains interpret visual and auditory signals.

**Related research article** Metzen M, Hofmann V, Chacron M. 2016. Neural correlations enable invariant coding and perception of natural stimuli. *eLife* 5:e12993. doi: 10.7554/eLife.12993

**Image** Simulation of the electric fields produced by two weakly electric fish in close proximity to each other

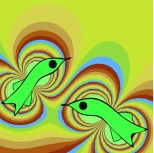

The question of how animals make sense of their environment is of interest in both neuroscience and computer science. The key challenge is to understand how to categorize events in ways that are useful to the animal, and also practical to implement. Typically, this involves separating information about an event (for example, what kind of object has just appeared in the animal's environment?) from information about where the event happened (DiCarlo et al., 2012).

While much of the research in this area has focused on vision, similar computational principles are involved for other senses, including smell and hearing (King and Nelken, 2009). In particular it is thought that the initial signal detected by sensory neurons in the peripheral regions of the brain undergoes a series of transformations as it is passed from one set of neurons to another. The end result is to produce an representation of the event (for example, the presence of another animal of a particular species) that does not depend on where the event happened. However, much remains to be understood about the transformations that would make this possible.

In this situation, studying species with exotic senses not used by humans might shed new light on the problem. Now, in eLife, Michael Metzen, Volker Hofmann and Maurice Chacron from McGill University report the results of experiments on fish called brown ghost knifefish (Metzen et al., 2016). These fish live in murky water and produce electric signals in order to “see” their environment. Such fish are known as weakly electric fish because the electrical discharges they produce, while strong enough to "see" objects, are not strong enough to be used as weapons.

A brown ghost knifefish emits an electric field that oscillates at a given frequency and creates an electric field that surrounds the fish. It also uses an array of electroreceptors on its surface to detect changes in this electric field: these changes could be caused by other fish, both electric and non-electric, predator or prey, as well as other objects. Metzen et al. focused on what happens when a knifefish communicates with another knifefish. Each knifefish emits at a slightly different frequency, so when two knifefish come into close proximity of each other, these frequencies interfere to generate a beat pattern. The frequency of this beat pattern is equal to the difference between the two original frequencies.

If a knifefish wants to communicate with another knifefish, it produces a “chirp” – a temporary increase in the frequency of its electric field discharge (Zupanc and Maler 1993; Figure 1). This leads to a change in both the frequency and phase of the beat pattern (with the change in the phase having any value between zero and 360 degrees). The phase can change again (by between zero and 360 degrees) depending upon the timing of the response from the second fish. Given this ambiguity, how can the fish recognize each other when each fish can produce a wide range of different phase changes in the beat pattern?

**Figure 1.**
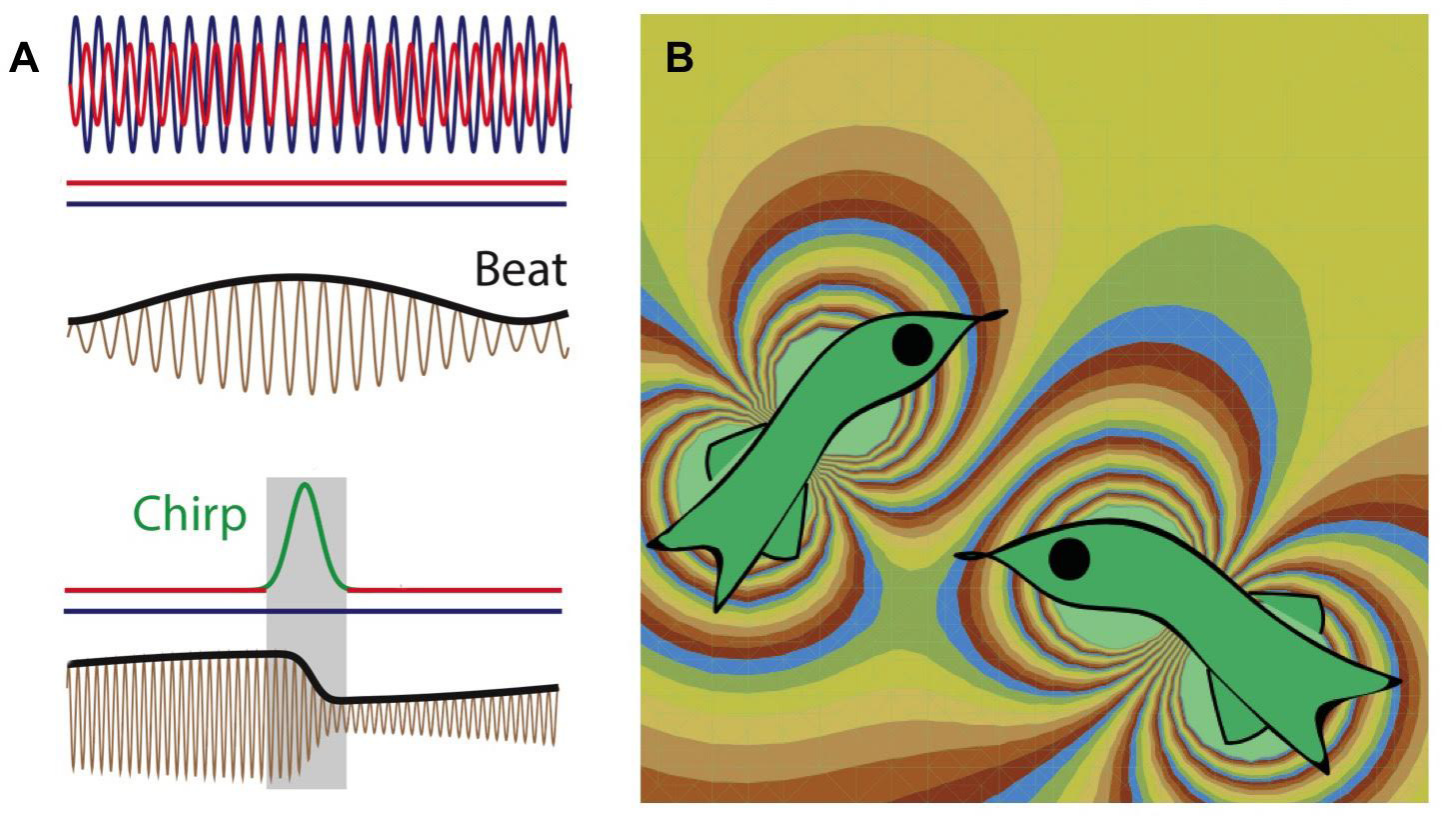
Weakly electric fish generate electric fields to communicate and to probe their environment. (**A**) The electric fields produced by two electric fish (red and blue lines; top) interfere to produce a beat pattern (brown and black lines; middle); the horizontal red and blue lines indicate that the frequency of each field does not change with time. If the red fish wants to communicate with the blue fish, it increases the frequency of the electric field it produces for a short time (red-green-red line; bottom). This "chirp" changes both the frequency and phase of the beat pattern (brown and black lines; bottom). Image from Metzen et al. (**B**) Computer simulation (computed using the dipole approximation) showing the electric fields produced by two electric fish. The fish on the left is able to detect the presence of the fish on the right because the latter changes the electric field in the vicinity of the former, and vice versa. The different colors represent different strengths and directions of the electric field.

Metzen et al. studied how the communication signal is represented in three successive stages of neural circuits: circuits formed by neurons that are directly connected to the electroreceptors on the surface of the fish; circuits formed by pyramidal neurons in the hindbrain; and circuits formed by neurons within the Torus semicircularis in the midbrain. Metzen et al. report that neural responses at each successive stage of processing depend less and less on the phase. This process of making the signals 'phase invariant' is similar to how visual signals are processed (Movshon et al., 1978; Sharpee et al., 2013).

Studies of the comparatively simple neural circuits of the weakly electric fish can also provide insights into the neural mechanisms behind invariant responses in other more complex sensory systems. For example, the responses of the individual peripheral neurons in the knifefish are not phase invariant: however, the activity of these neurons is correlated in a way that is phase invariant. This shows that one way to generate a phase-invariant response is to correlate and combine a number of neural responses.

A prominent aspect of electrosensation is that the animal has control over the types of signals that it produces. This makes it a rich ground for testing the theory that biological circuits are optimized to transmit maximal information for a given cost (Polani, 2009; Bialek, 2013). Relevant questions include: why are the electro-receptors on the surface of the fish distributed the way they are, and why do the communication calls used by knifefish have the structure they do? And given that fish continue to grow throughout their lives, how does the electrosensory system continue to operate when the anatomy of the fish is changing constantly? An answer to this last question could help to improve our understanding of how mammalian systems cope with aging and adaptation (Webster et al., 2006).

More generally, one expects to find many solutions to the problem of information optimization, depending on the characteristics of the environmental niche for a particular animal and the metabolic costs they can afford (Tishby and Polani, 2011). The diversity of these solutions can be mapped onto biological diversity. For example, why do some fish probe their environment with electric fields, whereas bats use echolocation? From a computational perspective, there are also parallels between electrosensation in weakly electric fish and song communication in birds, auditory vocalizations in dolphins and non-human primates, and human speech (Kanwal and Rauschecker, 2007). Comparative analysis of these systems in terms of information and energy efficiency will help to elucidate the general principles of how neural circuits work.

## References

[bib1] Tishby N, Polani D, Cutsuridis V, Hussain A, Taylor JG (2011). Information theory of decisions and actions. Perception-Action Cycle.

[bib2] Bialek W (2013). Biophysics Searching for Principles.

[bib3] DiCarlo JJ, Zoccolan D, Rust NC (2012). How does the brain solve visual object recognition?. Neuron.

[bib4] Kanwal JS, Rauschecker JP (2007). Auditory cortex of bats and primates: Managing species-specific calls for social communication. Frontiers in Bioscience.

[bib5] King AJ, Nelken I (2009). Unraveling the principles of auditory cortical processing: Can we learn from the visual system?. Nature Neuroscience.

[bib6] Metzen M, Hofmann V, Chacron M (2016). Neural correlations enable invariant coding and perception of natural stimuli. eLife.

[bib7] Movshon JA, Thompson ID, Tolhurst DJ (1978). Receptive field organization of complex cells in the cat's striate cortex. Journal of Physiology.

[bib8] Polani D (2009). Information: Currency of life?. HFSP Journal.

[bib9] Sharpee TO, Kouh M, Reynolds JH (2013). Trade-off between curvature tuning and position invariance in visual area V4. Proceedings of the National Academy of Sciences of the United States of America.

[bib10] Webster MA, Mizokami Y, Svec LA, Elliott SL (2006). Neural adjustments to chromatic blur. Spatial Vision.

[bib11] Zupanc GKH, Maler L (1993). Evoked chirping in the weakly electric fish *apteronotus leptorhynchus* : A quantitative biophysical analysis. Canadian Journal of Zoology.

